# Comparative domain modeling of human EGF-like module EMR2 and study of interaction of the fourth domain of EGF with chondroitin 4-sulphate^☆^

**DOI:** 10.1016/S1674-8301(11)60013-4

**Published:** 2011-03

**Authors:** Mukta Rani, Manas R. Dikhit, Ganesh C Sahoo, Pradeep Das

**Affiliations:** Biomedical Informatics Division, Rajendra Memorial Research Institute of Medical Sciences, Agam Kuan, Patna-800007, India

**Keywords:** EMR2, G-protein coupled receptor, transmembrane, homology modeling, EGF-TM7

## Abstract

EMR2 is an EGF-like module containing mucin-like hormone receptor-2 precursor, a G-protein coupled receptor (G-PCR). Mutation in EMR2 causes complicated disorders like polycystic kidney disease (PKD). The structure of EMR2 shows that the fifth domain is comprised of EGF-TM7 helices. Functional assignment of EMR2 by support vector machine (SVM) revealed that along with transporter activity, several novel functions are predicted. A twenty amino acid sequence “MGGRVFLVFLAFCVWLTLPG” acts as the signal peptide responsible for posttranslational transport. Eight amino acids are involved in N-glycosylation sites and two cleavage sites are Leu517 and Ser518 in EMR2. The residue Arg241 is responsible for interaction with glycosaminoglycan and chondroitin sulfate. On the basis of structure, function and ligand binding sites, competitive EMR2 inhibitors designed may decrease the rate of human diseases like Usher's syndrome, bilateral frontoparietal polymicrogyria and PKD.

## INTRODUCTION

G protein-coupled receptors (GPCRs) belong to the family of integral-membrane proteins (IMPs) that transduce external signals through the cell membrane[1] to regulatory G-proteins, which in turn trigger a wide range of biological events[2]. GPCRs are sensory proteins and important receivers of different external stimuli such as hormones, neurotransmitters, neuromodulators, odours and light. GPCRs represent the largest family of cell-surface receptor molecules that are involved in signal transmission, accounting for >2% of the cellular proteins encoded by the human genome and they are the targets for 50% of all recently launched drugs[3],[4]. GPCRs are characterized by the presence of highly conserved molecular architecture encoding seven transmembrane (TM) hydrophobic regions linked by three extracellular loops that alternate with three intracellular loops as confirmed by analysis of the crystal structure of rhodopsin[5]. The extracellular N-terminus is glycosylated, and the cytoplasmic C-terminus is generally phosphorylated. The GPCR family comprises the largest family of cell-surface receptors, which can sense information encoded by diverse external stimuli and translate the encoded information into readable signals for the cell[6].

EMR2 is an epidermal growth factor (EGF)-like module containing mucin-like hormone receptor-2 precursor and a cell surface protein receptor (GPCR) restricted to leukocytes and/or smooth muscle cells in humans[7]. These receptors are characterized by an unique hybrid structure consisting of tandem repeats of EGF-like modules. It is coupled to the N-terminal family B (secretin receptor) GPCR related to the seven- transmembrane (TM7) receptor domains by a glycosylated stalk region[8],[9]. EGF-like module EMR2 receptors is predominately expressed in cells of the immune system and binds to ligands such as CD312. The *EMR2* genes are predominantly expressed on dendritic cells, monocytes and macrophages. The EGF-TM7 molecules belong to a large protein family known as the long N-terminal of group B transmemberane 7 (LNB-TM7) or B2 subgroup of class B GPCRs with large extracellular N-terminal domains[8]–[9]. All LNB-TM7 receptors that possess large extracellular domains consisting of various protein modules are implicated in protein-protein interactions. Along with adhesion and signaling function, these molecules also participate in the interaction of the extracellular region with other cell surfaces or extracellular matrix proteins through the transmembrane domain[10]. LNB-TM7 receptors have different extracellular structural domains that are separated from the TM7 region by an extended spacer region. A subgroup of the LNB-TM7 receptors is the EGF-TM7 family[11]. Genomic mapping analysis has suggested a possible EGF-TM7 gene family on the human chromosome 19p13.1 region and possesses similar exon-intron organizations[11] or the LNB-TM7 receptors[8] that contain a large N-terminal cell adhesion-like extracellular domain coupled to a secretin receptor-like TM7 domain.

These EGF domains are coupled to TM7 via an extended spacer region. As a result of alternate RNA splicing, receptor isoforms possessing variable numbers of EGF domains are expressed. The EGF domains of EGF-TM7 receptors have been shown to mediate binding to cellular ligands. The domain 4 of EMR2 interacts with glycosaminoglycan (GAG) and chondroitin sulfate[7]. Ligand specificity for chondroitin sulfate is shared by EMR2, whose EGF domain region is highly similar to that of CD97. Only 6 out of 236 amino acids differ within the five EGF domains[9]. To date, four isoforms have been reported for EMR2, containing two (EGF1, 2), three (EGF1, 2, 5), four (EGF1, 2, 3, 5), or five (EGF1, 2, 3, 4, 5) EGF domains. Antibody-blocking studies subsequently revealed that the fourth domain of EGF-like module constitutes the major ligand-binding site[7]. The ligand for the largest isoform of EMR2 has recently been identified as chrondroitin sulphate, which binds to the EGF-like module of EMR2. It has been shown to interact with chondroitin sulfate and GAG in an isoform-specific manner[10].

The human-restricted adhesion-GPCR, EMR2, regulates neutrophil responses by potentiating the effects of a number of proinflammatory mediators and it has been shown that the transmembrane region is critical for adhesion-GPCR function. On neutrophil activation, EMR2 is rapidly translocated to membrane ruffles and the leading edge of the cell[11] and in monocytes and macrophages, EMR2 can be specifically up-regulated by lipopolysaccharides (LPS) and IL-10 via an IL-10-mediated pathway[10].

EMR2 is a myeloid cell-restricted member of the EGF-TM7 family that is closely related to CD97[8]–[9]. The EGF-like domains of the full-length EMR2 protein share 97.5% sequence identity with CD97. Similar to CD97, distinct EMR2 protein isoforms consisting of different numbers of the EGF-like domains have been documented[9]. Tissue specificity of EMR2 expression is highest in peripheral blood leukocytes, followed by spleen and lymph nodes, with intermediate to low levels in the thymus, bone marrow, fetal liver, placenta and lung. The extracellular domain of EGF-TM7 receptor consists of tandem repeats of EGF-like modules followed by a Ser/Thr-rich stalk and a GPS motif[8]. The GPS motif is primarily found in members of class B2 GPCRs. These include the human polycystic kidney disease protein (PKD)-1[12]–[13], suREJ3, a channel-like 11-span transmembrane protein[14] and hPKDREJ, the human homologue of suREJ3[15], suggesting that the GPS motif and its associated proteolytic cleavage activity are widely used by cell surface receptors. Although the functional significance of the GPS motif-associated proteolysis remains elusive, the presence of the highly conserved GPS motif in such a diverse array of receptors is suggestive of a common role in receptor function or regulation.

Mutations within this region of adhesion-GPCRs occur in a number of human diseases, including Usher's syndrome, bilateral frontoparietal polymicrogyria and PKD[16]–[18]. In PKD, cystic tubules are unable to perform this function properly, resulting in fluid retention, high blood pressure and kidney failure requiring dialysis or transplantation. In this study, we investigated the structure and protein interactions of EMR2 using bioinformatics tools.

## MATERIALS AND METHODS

### Structural modeling

The sequence of human EMR2 protein (823 amino acids) was retrieved from NCBI. Multiple alignments of the related sequences were performed using the Clustal W program accessible through the European Bioinformatics Institute (http://www.ebi.ac.uk/Tools/clustalw2/index.html)[19]. The tertiary structures of different domains of EMR2 were modeled on the basis of different template structures from MODELLER 9v6. Structure validation was performed by using 3-D molecular modeling tool Verify protein (DOPE score) of Discovery Studio v2.1 (Accelrys).

### Prediction of different domains of EMR2

Determination of various domains in EMR2 was carried out by using GPCRDB[20], DOMAC Protein Domain Prediction program[21] and SCRATCH program[22]. The GPCRDB is a database that collects molecular class-specific information system on GPCRs. DOMAC server is an accurate protein domain prediction server combining both template-based and *ab initio* methods. SCRATCH is a server for predicting protein tertiary structures that includes predictors for secondary structure, domains, disulfide bridges, single mutation stability and tertiary structure.

### Transmembrane region prediction

Different servers i.e. TMHMM, SOSUI, HMMTOP, TMpred, Das and TopPred servers were accessed to validate the TM region of EMR2[23]–[28].

### Protein function assignment of EMR2

We employed SVMProt server with support vector machine (SVM) learning techniques, which classifies a protein into functional families from its primary sequences[29], to identify novel functions of EMR2. Novel protein function assignments of different proteins of SARS virus, *Japanese encephalitis* virus and lipophosphoglycan 2 (LPG2) protein of different strains of *Leishmania* have already been reported using the technique[30]–[32].

### Ligand binding site prediction

Pocket-Finder or Q-site finder is a molecule-binding site prediction server based on Ligsite algorithm[33]. It works by scanning a probe radius 1.6A° along all gridlines of grid resolution 0.9A° surrounding the protein. The probe also scans cubic diagonals. Grid points are defined to be part of a site when the probe is within range of protein atoms followed by free space followed by protein atoms. Grid points are only retained if they are defined to be part of a site at least five times[33].

### Eukaryotic Linear Motif (ELM) server

Functional sites in eukaryotic proteins which fit the description “linear motif” are specified as patterns using regular expression rules. ELM server provides core functionality including filtering by cell compartment, phylogeny, globular domain class (using the SMART/Pfam databases) and structure[34]. Individual functions assigned to different sequence segments are combined to create a complex function for the whole protein.

### Protein-ligand interaction study

Protein-ligand interaction, i.e. docking, was studied by LigandFit/LigandScore[35] in DS Modeling 2.1. Interaction study with some other proteins like KMP-11 in *Leishmania* of six different strains has been reported[36]. LigandFit is an automated tool for docking/scoring study that includes the following protocols: 1) define binding site (ligand-based or cavity-based); 2) generate ligand conformations (Monte Carlo trials); 3) dock each conformation (align shapes of ligand to binding site; 24 orientations of ligand, rigid body energy minimization with grid-based energy function); 4) save the top docked structures (diverse poses); 5) apply scoring function(s) to each docked structure for the best binding mode (binding affinity prediction).

### Normal mode analysis of EMR2 3-D structure

Normal mode analysis was conducted to analyze the intrinsic motions of the EMR2 modeled structure. The elNémo online server (http://igs-server.cnrs-mrs.fr/elnemo/index.html)[38] was employed in our study for this purpose. This server is a part of the Elastic Network Model, which provides a fast simple tool to compute, visualize and analyze low-frequency normal modes of biological macromolecules. The structural model of EMR2 in dot pdb (.pdb) format was submitted for normal mode analysis while another 3-D structure complex was submitted as a reference of the desired conformational change. The key parameters which were used in computation included: DQMIN=-100, DQMAX=100, DQSTEP=20, and NRBL = “auto”. A total of 100 normal modes with the lowest frequencies were requested. The normal mode theory is based on the harmonic approximation of the potential energy function around a minimum energy conformation. This approximation allows an analytical solution of the motion by diagonalizing the Hessian matrix. Hessian matrices are used in large-scale optimization problems within Newton-type methods because they are the coefficient of the quadratic term of a local Taylor expansion of a function. That is: 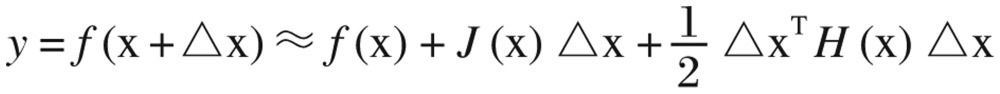 where *J* is the Jacobian matrix, which is a vector (the gradient) for scalar-valued functions. The full Hessian matrix can be difficult to compute in practice; in such situations, quasi-Newton algorithms have been developed that use approximations to the Hessian. The most well-known quasi-Newton algorithm is the BFGS algorithm[39],[40].

## RESULTS AND DISCUSSION

### Different domains of EMR2

Five EGF calcium binding domains are predicted in the sequence of EGF-like module EMR2 by using three different servers i.e. GPCRDB, DOMAC program and SCRATCH program. The first domain of EMR2 consists of 68 amino acid residues (1-68); similarly, the second domain consists of 69-120 residues, the third domain lies in between amino acids 121-238 and the fourth and fifth domains stretch out in amino acids 239-318 and 319-823, respectively. The GPCR proteolytic sites (GPS) occur in amino acid residues 479-529. The folding pattern of this protein consists of double-stranded β-sheet followed by a loop to a C-terminal short double-stranded sheet.

**Fig. 1:**
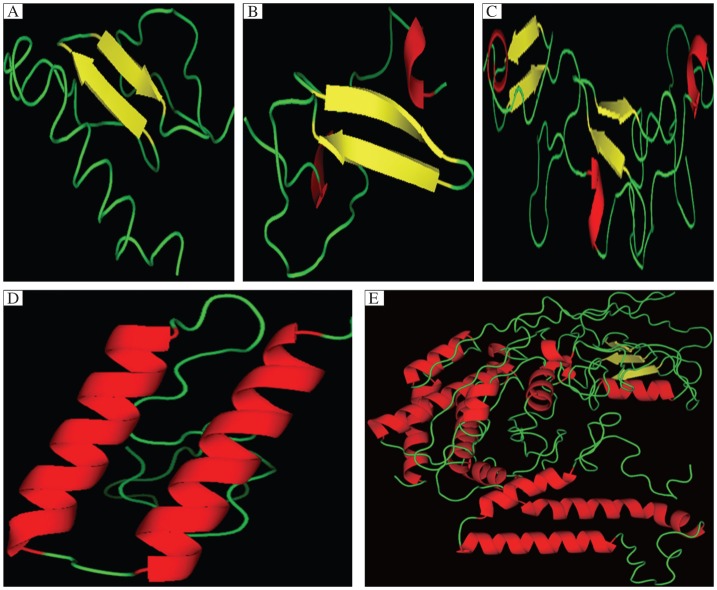
Ribbon representation of the modeled EMR2 protein of the entire five different domains. The image was created using Discovery studio (Accelrys) software. A: Domain 1 consists of an antiparallel β-sheet. B: Domain 2 consists of 1 antiparallel β-sheet and 2 small helices. C: Domain 3 consists of 2 antiparallel β-strands, and 3 small helices. D: Domain 4 consists of only 2 α-helices. E: Domain 5 consists of 7 transmembrane helices and 3 parallel β-strands and 11 small α-helices.

Modeler program constructed the structural models of full length EMR2 considering different templates i.e. EGF domain 1, 2, and 5 of human EMR2, a 7-TM immune system molecule in complex with barium (pdb id: 2BOU) and EGF domain 1, 2, and 5 of human EMR2, a 7-TM immune system molecule in complex with calcium (pdb id: 2BO2). The first domain 3-D structure was constructed by using three different templates i.e. EGF domain 1, 2, and 5 of human EMR2 with barium (pdb id: 2BO2), EGF-like module of human C1R (pdb id: 1APQ) and fibrillin-1CB EGF protein (pdb id: 1LMJ_A). The verify protein (DS, Accelrys, USA) score of the 1^st^ domain structure of EMR2 is 15.97 and the 2^nd^ domain is 15.99. No invalid region was found in the model. Similarly, the three dimensional model of the 3^rd^ domain was modeled with templates named human notch-1 ligand binding protein (pdb id: 1TOZ_A) and EGF domain 1, 2, and 5 of human EMR2, a 7-TM immune system molecule (pdb id: 2BOU) and showed high DOPE score. The three dimensional structure of the 4^th^ domain of EGF-like module EMR2 showed good DOPE score i.e. 35.88. Dali server[38] was accessed to compare template structures of this domain i.e. the crystal structure of phosphoribosyl-ATP pyrophosphatase (pdb id: 1YXB) and four helix bundles (pdb id: 1JMO). The three dimensional structure of the 5^th^ domain of EMR2 was modeled by using structural coordinates of six PDB structures i.e. hemochromatosis protein HFE complexed with transferin (pdb id: 1DE4), bovine rhodopsin (pdb id: 1U19), prostate-specific membrane antigen A (pdb id: 1Z8L), bovine rhodopsin (pdb id: 1L9H), peroxisomal acyl-coA oxidase-II (pdb id: 1IS2), and adaptor protein 2 clathrin adaptor core (pdb id: 1GW5) with a DOPE score of 124.83. The modeled structure of five different domains of EMR2 is shown in ***Fig. 1***. RMSD values of different models were found to be within limits.

To validate the models, we further evaluated three dimensional structures of EGF-like module EMR2 with Ramachandran plot. Stereo-chemical evaluation of backbone Psi(Ψ) and Phi (Φ) dihedral angles of five modeled EMR2 domains was revealed in different percentages, i.e. 72%-90%, 3%-31% and 2%-14% residues fell within the most favored regions, additionally allowed regions and generously allowed regions and few residues are in disallowed region of Ramachandran plot, respectively (***supplementary Fig. 1***). The model has a normal distribution of residue types over the inside and the outside of the protein.

**Fig. 2:**
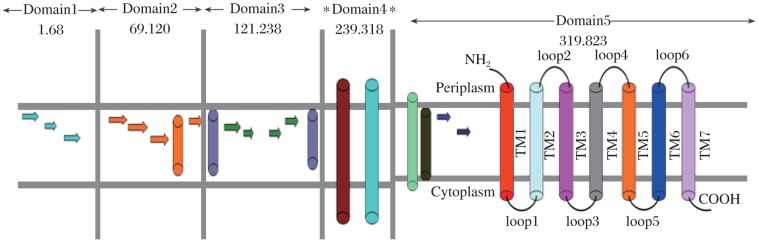
Schematic representation of modeled 3-D structure. It shows the five EGF calcium binding domains present in EGF-like module EMR2. Arrows represent helices in all domains in different colors and cylindrical represents the helix. Seven transmembrane helices are predicted in the fifth domain of EMR2. The amino acid residues vary in the first domain (1-68), second (69-120), third (121-238), fourth (239-318) and fifth domain (319-823). There are six extracellular loops that connect the seven transmembrane helices.

Five structural domains (1-5; NH_2_ to COOH terminus) are found to be present in EMR2. The first domain is composed of 68 amino acids and is hydrophilic in nature. Two anti-parallel β-sheet structures have been found in the first domain of EMR2. The second domain is comprised of 51 amino acid residues, and contains one anti-parallel β- sheet and two small helical regions. The third domain of EMR2 is comprised of 117 amino acids and consists of two anti-parallel β-sheets and three small helical regions are shown in ***Fig. 1***. The fourth domain contains 79 amino acids residues, which contribute to the helix-loop-helix domain, and it consists of only two α-helices. The C-terminal domain, i.e. the fifth domain of EMR2, consists of 508 amino acid residues. It consists of three parallel β-sheets, seven transmembrane helices (TMHs) and 11 small α-helices. Domain-wise structural configurations of EMR2 of all five different domains are shown in ***Fig. 2***.

### Sequence analysis of EMR2

Amino acid sequence of EMR2 (NCBI, gi108935835) was downloaded and aligned with six different genes (gi23397681, gi23397685, gi23397687, gi23397689, gi23397693 and gi23397691), which showed close identity. From the 1^st^ to 118^th^, 261^st^ to 398^th^ and from 410^th^ to 823^rd^ amino acids of EGF-like module containing mucin-like, and hormone receptor-like sequence 2 isoforms (of *Homo sapiens*) are identical to six other EMR2 sequences (gene id: gi23397681, gi23397685, gi23397687, gi23397689, gi23397693 and gi23397691). Non-synonymous mutations are deleterious in nature[42]. Various types of mutations have been observed to occur in this human protein, among which deletion or insertion at many regions of this EMR2 like protein has been detected. Two amino acids “DV”, which are likely to be present at position 119-120, are absent in the strain (gi23397687). These two amino acids may be insertion type mutations for other related strains, but for this strain it is a deletion type mutation.

### Transmembrane helices of EMR2

Different transmembrane prediction programs i.e. DAS, HMMTOP, TMHMM, TMpred, TopPred and SOSUI (***Table 1***) predicted the position and number of transmembrane regions in EMR2 amino acid sequence (***Fig. 2E***). The comparative analyses of four transmembrane prediction programs i.e. DAS, HMMTOP, TMHMM and TMpred showed that the 536^th^-561^st^ amino acids (538^th^-560^th^ in few cases) are responsible for the formation of the first transmembrane region. Similarly, the 571^st^-591^st^ and 600^th^-626^th^ amino acids of the fifth domain participate in formation of the second and third transmembrane regions, respectively. Other amino acids 646-649, 688-710, 730-756 and 758-782 are involved in constructing the 4^th^, 5^th^, 6^th^, and 7^th^ transmembrane helices of EMR2, respectively.

**Table 1:** Predicted locations of the transmembrane helices (TMs) on EGF like EMR2 protein in human

Methods	N^a^	TM1	TM2	TM3	TM4	TM5	TM6	TM7	TM8
DAS	7	—	538-560(20)^b^	575-589(20)	600-626(26)	654-659(20)	688-709(20)	738-749(20)	760-781(20)
HMMTOP	7	—	536-560(20)	573-590(20)	601-625(25)	646-664(25)	685-709(20)	730-749(25)	758-782(25)
TMHMM	7	—	538-560(22)	571-589(25)	608-626(25)	646-664(25)	683-705(25)	738-756(20)	760-782(25)
TMpred	7	—	543-561(20)	573-591(25)	609-629(25)	646-665(25)	688-710(25)	734-753(25)	758-777(25)
TopPred	8	395-415(20)	541-561(25)	571-591(25)	599-619(25)	645-665(25)	690-710(25)	734-754(22)	761-781(25)
SOSUI	7	—	538-560(25)	538-560(25)	601-623(22)	646-667(25)	690-711(21)	734-756(25)	762-784(25)

^a^ predicted number of TMs; ^b^ Numbers in brackets are the lengths of TMs

The disulfide bond formation in different domains of EMR2 (Dipro/ SCRATCH protein predictor program) is shown in ***supplementary Table 1***. The amino acids of the first domain are found to be involved in formation of disulfide bond at three different sites, 29-39, 33-45 and 47-65, respectively. The amino acids of the second domain of EMR2 are also involved in three disulfide bonds in regions 71-85, 79-94 and 96-117, respectively. Seven disulfide bond formation sites are found in the third domain of EMR2 and are found in residues 123-136, 130-145, 147-161, 167-180, 174-189, 191-210 and 216-229. Only one disulfide bond on residues 240-259 is present in the 4^th^ domain of EMR2. The last domain of EMR2 has five disulfide bonds i.e. 482-500, 492-514, 561-599, 676-693 and 743-746, respectively.

### Posttranslational modification

**Fig. 3:**
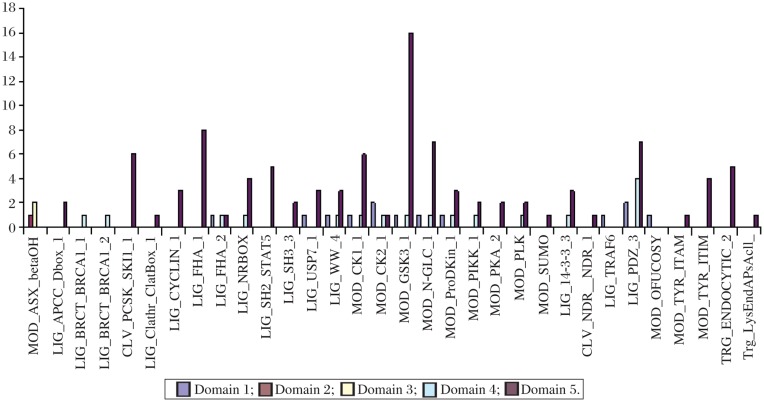
ELM server graph. The different motifs are present in five different domains of EMR2 predicted through ELM server.

Posttranslational modifications are important for future localization or function of the protein. Various posttranslational modification sites have been identified in the EGF-like module protein EMR2 (***Fig. 3***). A signal peptide is found in the first twenty amino acids (amino acid sequence: MGGRVFLVFLAFCVWLTLPG) in EMR2 as predicted from SOSUI signal server. In the 2^nd^ and 3^rd^ domains, EGF has posttranslational ASX hydroxylation at the conserved aspartate or asparagine residues, forming erythro-β-hydroxyaspartic acid or erythro-β-hydroxyasparagine. In the fifth domain, APCC-binding destruction motifs are present. The BRCT domain of the fourth domain is associated with DNA damage response and recognizes and binds to specific phosphorylated serine sequences. PCSK cleavage site motif is present in the fifth domain of EMR2. A cyclin recognition motif that interacts with cyclin has also been detected in domain 5. FHA phosphopeptide ligand motif 1 is present in domain 5, and motif 2 is present in domain 1, 4 and 5. The FHA domain is a signal transduction module, which recognizes phosphothreonine-containing peptides on the ligand proteins. In domain 1, 4 and 5, ww ligand motifs are predicted that are implicated in protein-protein interactions mediated by WW domains. Casein kinase (CK1) phosphorylation motifs (recognized by CK1 and CK2 for Ser/Thr phosphorylation) are present in domain 1, 4 and 5. In domain 1, 4 and 5, GSK3 phosphorylation site is present and in domain 5, and 16 different patterns of this motif are present and shown in ***Fig. 3***. GSK3 comprised of two highly related proteins (GSK3-α and GSK3-β) phosphorylates a wide variety of target proteins. N-glycosylation, a posttranslational process involving the transfer of an oligosaccharide chain to asparagine residue in the protein, is found in domain 1, 4 and 5. MAPK phosphorylation, which pro-directs kinases such as P38 MAP kinase to phosphorylate Ser/Thr in various signal transduction pathways. Domain 4 and 5 contain the phosphorylation motifs of phosphoinositide-3-OH-kinase related kinases (PI3KKs), which are atypical protein kinases exclusive to eukaryotes.

### Ligand binding sites of EMR2

The potential ligand binding sites of the five domains of EMR2 have been found by Pocket Finder program. A total of ten possible binding sites were obtained in the third, fourth and fifth domain. Different ligand binding sites of the five different domains of EMR2 are shown in ***supplementary Fig. 2***. The frequently involved amino acid residues in forming the pocket are Try8, Thr27, Phe45, Cys49, His56, Thr64, Ile65, Leu76, Gln77, Thr113, Ser230, Ser232, Phe243, Val273, Tyr295, Thr300, Try301, Ala313, Ala341, Val377, Leu399, Thr420, Phe424, Ile425, Ile434, Ile450, Gln455, Try477, His491 and Thr501.

**Table 2:** Comparative analysis of EMR2 functional assignment of all the five different domains

Function from NCBI	Domain 1	Domain 2	Domain 3	Domain 4	Domain 5
Transmembranes					98.6%
l.Posttranslational modification 2.Protein turnover		EC 3.6.-.-: Hydrolases - Acting on acid anhydrides (58.6%) TC 3.A.1 ATP-binding cassette (ABC) family (58.6%)		EC 3.6.-.-: Hydrolases - Acting on acid anhydrides (58.6%) TC 3.A.1 ATP-binding cassette (ABC) family (58.6%)	
Metal-binding	58.6%	58.6%	58.6%	58.6%	58.6%
Metabotropic glutamate family					7 transmembrane receptor (58.6%)
Chaperones / Intracellular trafficking					G protein coupled receptors (99.2%)
Secretion					7 transmembrane receptor (58.6%) TC 3.A.5 Type II (general) secretory pathway (IISP) family (58.6%)
Copper binding	58.6%	58.6%	58.6%	58.6%	
Zinc binding	58.6%	58.6%	58.6%	58.6%	58.6%
All lipid-binding proteins	58.6%		58.6%		
Actin binding	58.6%		58.6%		

### Functional assignment of EMR2 by SVM

Different unknown and hidden functions of a protein (EMR2) were predicted using machine learning technique like a statistical SVM based classifier, the SVMProt (***Table 2***). Different domains of EMR2 were assigned to metal binding functional family by SVMProt, which correlates to previously published data that EMR2-chondroitin sulfate interaction is Ca^2+^ and sulphate ion-dependent, and results in cell attachment[7]. Comparative analyses have shown that different domains of EMR2 belong to the GPCR family. The 2^nd^ and 4^th^ domains are probably responsible for ATP-binding cassette (ABC) function.

**Table 3:** The 10 normal modes of EMR2 predicted by normal mode analysis method by the elNémo server

Mode^a^	Frequency	Collectivity^b^	Cumulative Overlap^c^	Amplitude (dq)
Mode7	1.00	0.3865	0.168	-1588.2302
Mode8	1.47	0.5461	0.183	-519.6375
Mode9	2.56	0.5447	0.244	927.5116
Mode10	3.38	0.5712	0.321	1066.0092
Model 1	4.12	0.4854	0.321	-199.8678
Mode12	5.38	0.5675	0.361	-791.8320
Mode13	5.92	0.4092	0.468	1253.7680
Mode14	6.92	0.2804	0.484	-468.0940
Mode15	7.08	0.5000	0.484	-189.8256
Mode16	7.79	0.3995	0.499	-422.8200

^a^: Only the 10 normal modes with lowest frequencies are displayed here. ^b^: The level of collectivity indicates the percentage of residues that are involved in a certain normal mode. ^c^: The level of overlap measures the similarity between a desired conformational change and that of a certain normal mode.

### N-glycosylation site in EMR2

**Fig. 4:**
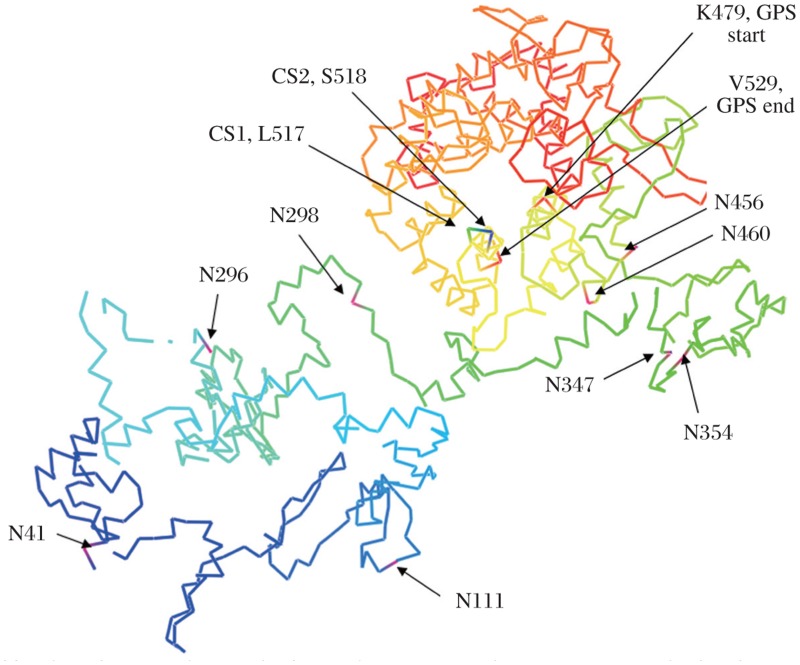
Different motifs showing N-glycosylation, cleavage and GPCR proteolytic sites. In human EMR2, a 7-TM immune system molecule, are shown A: N-glycosylation sites: 8 glycosylation sites are indicated by black arrows and highlighted in pink are N41, N111, N206, N298, N347, N354, N456 and N460; B: Cleavage sites (CS1 and CS2): two cleavage sites at L517 and S518 are highlighted by green and blue, respectively. C: GPCR proteolytic sites (GPS): the GPS site starts with K479 and ends with V529 and in total includes 50 amino acids (from the start and end of the region) and is shown in red.

The three dimensional model of EMR2 shows eight potential N-glycosylation sites. The eight amino acid residues involved in N-glycosylation sites are N41, N111, N206, N298, N347, N354, N456 and N460 (***Fig. 4***). Two cleavage sites were found in EMR2 at residues Leu517 and Ser518[43]. The GPCR proteolytic site starts with Lys479 and ends with Val529, and the GPCR proteolytic site of EMR2 involves 30 amino acids (***Fig. 4***).

### Interaction of the 4^th^ domain of EMR2 with chondroitin 4-sulfate

The fourth EGF domain of CD312 interacts with GAG chondroitin 4-sulphate where the ligand is specifically found on B cells from peripheral blood, on activated lymphocytes and myeloid cells. The helix-loop-helix domain of EMR2 interacts with the GAG; chondroitin sulfate is one of the proteoglycans. EMR2 does not interact with the ligand decay accelerating factor (CD55) for complement, unlike the related CD97 antigen, and indicates that these very closely related proteins likely have non-redundant functions. The *EMR2* gene produces multiple transcripts encoding distinct isoforms[9]. The chondroitin chains are present near the junctions between both of the NH_2_ and COOH-terminal globular domains and the central region[44]. In addition, only one potential N-glycosylation site in EMR2 is present in these regions. A modeled 3-D structure of the fourth domain of EMR2 was docked with a single strand of chondroitin 4-sulphate where the molecular mass is 17 kDa. The 3-D structure of chondroitin 4- sulphate was drawn in Chemsketch as *.mol file and converted into *.pdb format. The structure also identifies the specific interaction sites between EMR2 and C4-S molecule. Similarly, the crystal structure of human cathepsin K interacts with chondroitin 4-sulphate has already been reported[45].

**Fig. 5:**
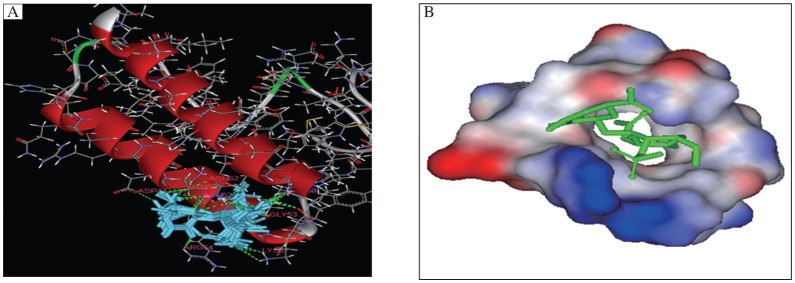
Interaction of the 4^th^ domain of EMR2 with chondroitin 4-sulfate. A: A screenshot from interaction with chondroitin 4-sulfate of domain 4 of EMR2. Ten different binding poses of chondroitin 4-sulfate are shown with the interaction or dock score between 31.607 and 81.147. The protein-ligand interaction is done through ligand receptor interaction protocol i.e. Ligandfit of Discovery Studio 2.1 (Accelrys). It shows hydrogen-bonded ion-pair interaction formed between different atoms of the ligand and amino acids of EMR2 (Ser1, Arg3, Ser29, Asp47, Gln50, Gly53, Arg54, Tyr56, Lys57, Pro58, and Asn62). B: A surface representation of EMR2 protein showing how the molecule is divided into a positively-charged region rich in basic residues (blue) and a negatively charged region that has negatively-charged acidic groups (Asp and Glu). The negatively charged C4-S hexasaccharide (green color) is electrostatically attracted to the positively charged region of EMR2 from the active site.

It is known from the literature that the fourth domain of EMR2 has three active sites. Chondroitin 4-sulphate molecules were docked in a groove of EMR2 that is on the active site. Higher ligand protein interaction has been detected up to 81.147 dock score. Ten different binding conformations have been observed during docking study in DS (Accelrys). The groove contains several positively charged side chains that interact directly with the negatively charged groups on chondroitin 4-sulphate (***Fig. 5B***). Different interactions between chondroitin 4-sulphate hexasaccharide and EMR2 are shown in ***Fig. 5A***. Frequent H-bond formation has also been detected and the amino acids involved are Ser1, Arg3, Arg7, Ser29, Asp47, Gln50, Gly53, Arg54, Tyr56, Lys57, Pro58 and Asn62. Arg3 (the exact location in the whole sequence is Arg241) provides a hydrogen-bonded ion-pair interaction to the 4-sulfate group of O9, O10, O11 and O14 in ten different conformations. The next residue on the helical turn is Ser1 (the exact location in the whole sequence is Ser239) that interacts with O3, O8, O9, O12, and O11 in ten different conformations. In case of chondroitin sulphate, there are five amino acid residues Ser29, Gln50, Arg54, Pro58 and Asn62; all have direct interactions with groups on the hexasaccharide of chondroitin 4- sulphate, either via the side chains or the main chain peptide bonds of all ten different conformations (***Fig. 5A***).

It is well known that hydrogen bond plays an important role for the structure and function of biological molecules[45]. Five important amino acids Ser29, Arg3, Arg5, Gly53 and Asn62 (the exact locations are. Ser239, Arg241, Arg243, Gln292, and Asn301) are responsible for interaction with chondroitin 4-sulfate. Competitive inhibition of EMR2 by analogues of chondroitin 4-sulfate could help in lessening genetic disease burden, e.g. Usher's syndrome, bilateral frontoparietal polymicrogyria, and PKD.

### Normal mode analysis of EMR2 3-D structure

**Fig. 6:**
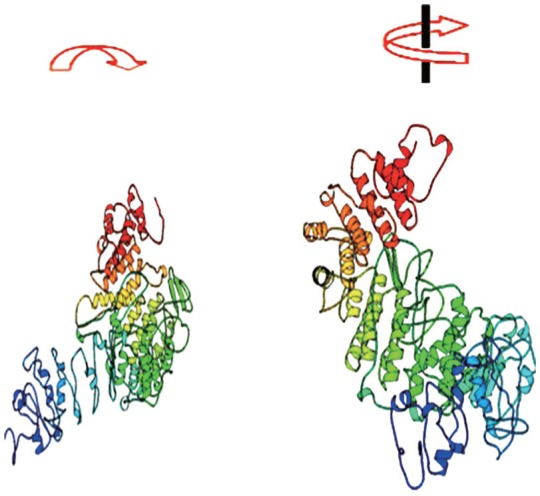
elNémo models. The two normal modes (i.e., mode 7 and 8) of EMR2 with the lowest frequencies (Left: bending of the N-terminal domain toward the transmembrane domain. Right: rotation of the N-terminal domain on the top of the transmembrane domain).

Normal Mode Analysis (NMA) program was run to know the native motions (vibrational and thermal properties) of the EMR2 modeled structure. Essential features of the top ten low-frequency normal modes, including its frequency, amplitude, collectivity of atom movements, and the overlap with observed conformational changes are summarized in ***Table 3***. The two lowest-frequency normal modes (mode 7 and 8 in ***Table 3***) are illustrated in ***Fig. 6***, one feature showing bending of the N-terminal domain towards the transmembrane domain; the other feature showing rotation of the N-terminal domain on the top of the transmembrane domain.

## Conclusion

On the basis of modeled structure, predicted binding sites and functions of EMR2, high throughput screening of various compounds may be carried out to find appropriate leads against different diseases, which can further be screened *in vitro* and *in vivo*.

## Figures and Tables

**Supplementary Table 1:** Different amino acid residues of disulphide bridges are comprised of five different domains of human EMR2 predicted by the DIpro (SCRATCH protein predictor) server.

Disulphide sites	Domain 1	Domain 2	Domain 3	Domain 4	Domain 5
Site 1	29-39	71-85	123-136	240-259	482-500
Site 2	33-45	79-94	130-145		492-514
Site 3	47-65	96-117	147-161		561-599
Site 4			167-180		676-693
Site 5			174-189		743-746
Site 6			191-210		
Site 7			216-229		

## Supplementary Materials

Supplementary Fig. 1 Ramachandran plot analysis. Ramachandran plot analysis of the five different domains of modeled structure of EMR2 like 2a(A), 2b(B), 2c(C), 2d(D) and 2e(E), respectively.

Supplementary Fig. 2 Binding site prediction. Prediction of ligand binding sites (LBSs) of all five different domains of EMR2 (A, B, C, D and E) in human by the pocket finder web server.
