# Supplementary figures and images for: Comparative domain modeling of human EGF-like module EMR2 and study of interaction of the fourth domain of EGF with chondroitin 4-sulphate
Source: J Biomed Res. 2011 Mar;25(2):100–10. doi: 10.1016/S1674-8301(11)60013-4 (PMC3596701; doi:10.1016/S1674-8301(11)60013-4)

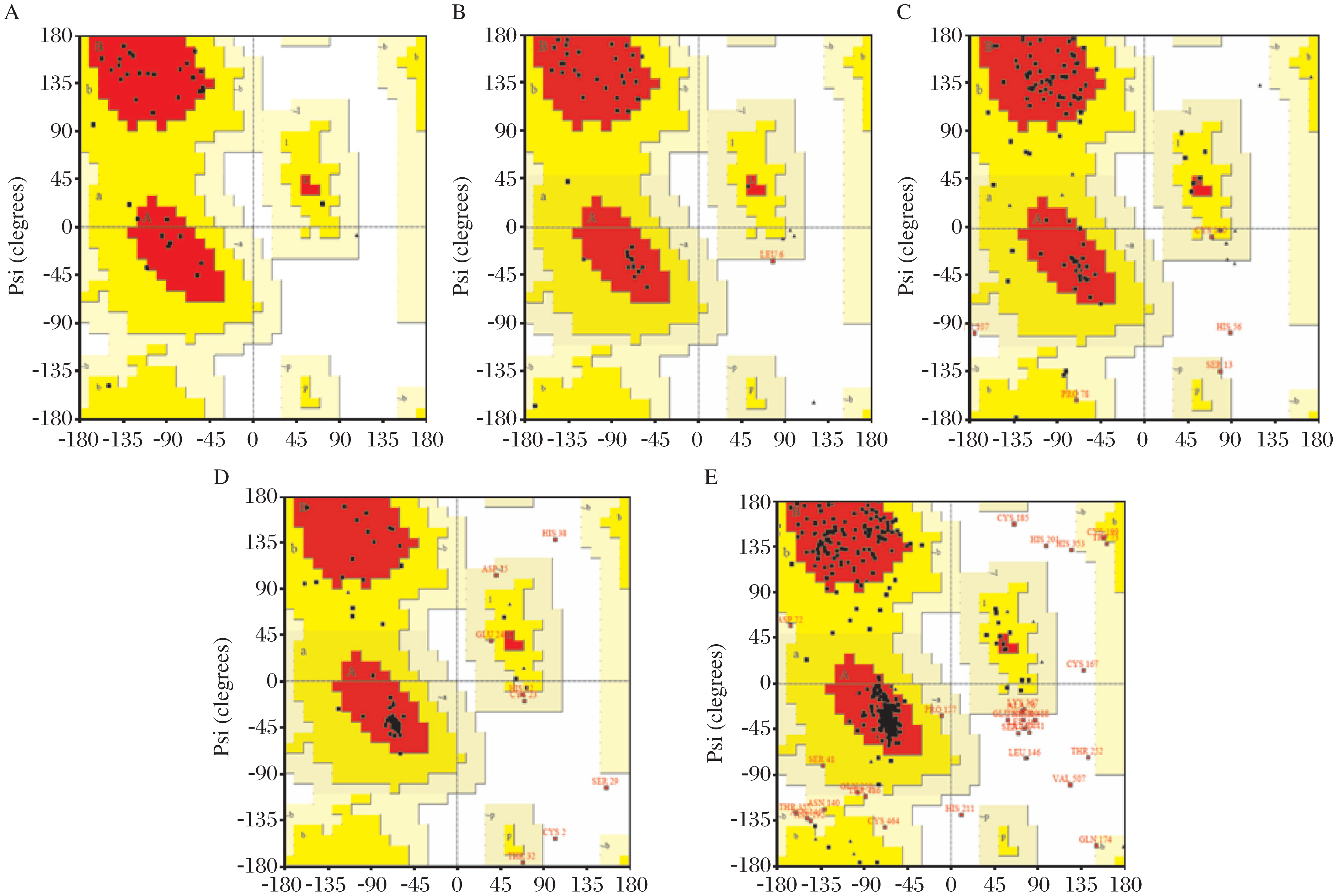

Supplement: Supplementary Fig. 1 — Ramachandran plot analysis of the five different domains of modeled structure of EMR2 like 2a(A), 2b(B), 2c(C), 2d(D) and 2e(E), respectively. [file jbr-25-02-100-s001.tif]

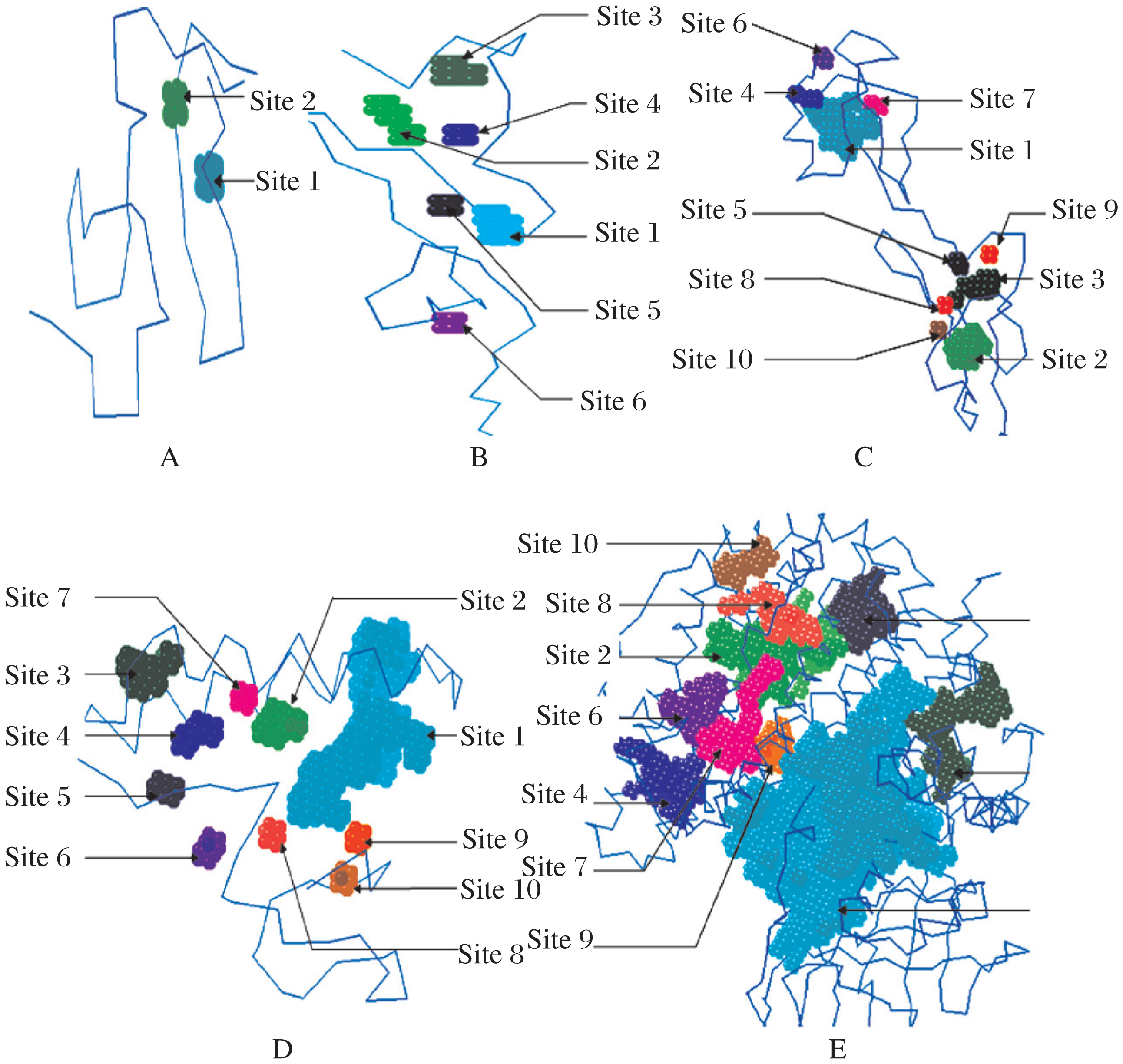

Supplement: Supplementary Fig. 2 — Prediction of ligand binding sites (LBSs) of all five different domains of EMR2 (A, B, C, D and E) in human by the pocket finder web server. [file jbr-25-02-100-s002.tif]
